# Expectations of manufacturing companies regarding future priorities of improvement actions taken by their suppliers

**DOI:** 10.1007/s12063-022-00307-2

**Published:** 2022-08-05

**Authors:** Maciej Urbaniak, Piotr Rogala, Piotr Kafel

**Affiliations:** 1 University of Lodz, Lodz, Poland; 2 Wroclaw University of Economics and Business, Wroclaw, Poland; 3 Cracow University of Economics, Kraków, Poland

**Keywords:** Supplier relations, Supply chain management, B2B market, Quality management, Process improvement

## Abstract

**Purpose:**

The paper concerns supply chains. It aims to identify the essential improvement actions that manufacturing companies expect their suppliers to take in the future rather than selection requirements already used in the assessment process. Within those improvement actions, the goal is to determine whether the size and management methods used in assessing companies affect these future expectations.

**Design/methodology/approach:**

Hypotheses were developed regarding the expectations of manufacturing companies and the factors influencing these expectations. Data collected from 118 companies from the Polish automotive, metal and chemical sectors were used to verify them. For this purpose, the U-Mann Whitney test was applied.

**Findings:**

Manufacturing companies expect their suppliers to improve: (1) products, (2) processes, (3) organization management, (4) communication and (5) relationship, with the most important thing for them is the improvement of products and processes. It was found that the expectations regarding the contributors are influenced by whether the manufacturing company is a large or small enterprise or it uses or not the Toyota Production System. Lean Management and the ISO 14,001 Environmental Management System do not translate into these expectations.

**Originality/value:**

: The article gives a new look at shaping relations between suppliers and customers in supply chains. Unlike most of the research to date, it does not concern the requirements that suppliers should meet at a given moment but focuses on the improvement actions they should undertake in the future. This paper adds important detail to understanding cooperation in B2B relations.

## Introduction

Achieving a competitive advantage requires that partners operating in the supply chains take improvement actions (Nikookar et al. 2021). This is particularly noticed by enterprises operating in production sectors, for which the competitive advantage is achieved mainly by improving the quality of products and by improving the effectiveness and efficiency of processes (Afraz et al. 2021).

To achieve a competitive advantage, many companies implement quality, environment and safety management systems as well as the concept of Toyota Production System or Lean Management projects (Islam 2019; Jayaram et al. 2010). When taking actions to improve processes and products, these enterprises also expect the implementation of improvement actions by suppliers. Clients on the B2B market, when making the initial assessment and selecting suppliers, often analyze potential threats. When assessing these threats, buyers also analyze the possibilities of improvement and development by suppliers (Basu et al. 2018; Lou et al. 2022). Initial evaluation and selection of suppliers is the basis for their qualification. A positive qualification status should ensure a low level of purchasing risk as well as guarantee opportunities for improvement and development by suppliers. For the selection process to be carried out effectively, buyers should define the prerequisites for suppliers in detail. These requirements are often published in the form of Supplier Guidelines, e.g. (Epson, Mahle), Supplier Handbooks (e.g. General Electric, Osram Sylvania, Avnet, Cummins, Knoll, Lilium), or Supplier Manuals (e.g. ABB, Airbus, BSH, Faurecia, First Solar, Kimball, Komatsu, Koni, Mercedes-Benz Nemak, Thyssen Krupp, Volvo). These requirements relate to both products (technical quality parameters, environmental performance) and processes (technology, efficiency, innovation). Buyers’ requirements also increasingly relate to the implementation of the concept of corporate social responsibility by suppliers based on the principles of Global Compact focusing on respect for human rights, ensuring labor standards, environmental protection and counteracting corruption (Gelderman et al. 2021; Tong et al. 2018). Recently, the sustainability and environmental goals aspects gain more attention within the supply chains (Rashid Khan et al. 2021). According to Awan et al. (2021) green innovation is becoming an imperative and instrumental source of environmental sustainability.

Verification of customer requirements is carried out through offer analysis, self-assessment questionnaires, audits, batch testing of products (Liou et al. 2021). Supplier evaluation is also carried out by examining the economic and legal situation of potential partners through due diligence (Taherdoost and Brard 2019). Audits are of particular importance in evaluating suppliers, as they allow to verify the accuracy of the information contained in the self-assessment questionnaires (Chen and Jeter 2008; Nikoofal and Gümüş 2020). When assessing during audits, customers mainly pay attention to the implementation of operational processes by suppliers, such as: customer service (accepting orders, handling complaints), research and development, production planning, production preparation, product quality control, process quality control, packaging, storage, product shipment or after-sales service. During audits, particular attention is paid to the documentation used (procedures and instructions and records of processes). Documented records from the control of processes and products (materials, semi-finished products, finished products) are vital in this assessment. During audits, customers more and more often focus on Safety-health and working conditions, as well as reducing the negative impact on the environment (Zakeri et al. 2022). After the evaluation and selection, the suppliers receive the qualification status. A positive qualification status enables suppliers to establish purchasing cooperation with customers. Customers entering into cooperation with qualified suppliers constantly monitor and periodically evaluate them. This evaluation is based on strictly defined criteria. The criteria that buyers use when assessing the supplier periodically include the level of technical quality offered, favorable price conditions, timely deliveries, attractive payment terms, having a quality system, the level of service quality, responses to complaints (Zakeri et al. 2022). This evaluation is carried out by means of pointers or scoring methods included in the supplier scorecard. Increasingly, this evaluation also includes improvement activities carried out by suppliers. Buyers set suppliers periodic targets for product and process improvement. These goals relate to improving the technical quality of products (reducing the level of non-compliance, implementing innovations), processes (shortening the implementation time, avoiding errors, increasing the level of security, ensuring continuity of implementation, improving communication, reducing costs) (Zhou et al. 2021). Suppliers are required to report (through self-assessment) the achieved results of the degree of compliance with their goals in Performance Feedback Reports Cards. This self-assessment is then verified through audits. It should also be noted that buyers may require an audit of the supplier in the event of non-compliance resulting in quality complaints or delays (e.g. due to disruptions in operational processes resulting in major failures or low production efficiency). Increasingly, buyers are setting supplier targets that focus on reducing the negative impact on the environment (Liou et al. 2019). This is related to the implementation of a sustainable supply chain concept (Azadi et al. 2015).

Much research has already been conducted to analyse the B2B market relationships between suppliers and customers. The analysis covered, among other things, critical success factors in supply chains (Eid et al. 2002; Kian Chong et al. 2011), ways to develop cooperation (Maestrini et al. 2018), factors affecting buying companies satisfaction (Mittal et al. 2021), the impact of satisfaction or dissatisfaction with the supplier on the actions taken by the buyer (André Mendes Primo et al. 2007; Suh and Kim 2018) as well as expectations of buying and selling companies (Kaski et al. 2017). However, in these studies, very little attention was paid to identifying the improvement actions that manufacturing companies expect their suppliers should undertake. Usually, even when issues related to improvement were included, they were among the many different factors taken into account (Sila et al. 2006), or they related to the need for improvement in general (Sharma 2021). There is clearly a lack of research that comprehensively covers the expectations of manufacturing companies regarding improvement activities undertaken by their suppliers. Due to the high pace of social and technological changes, deliveries on the B2B market cannot be limited only to the requirements that apply to them at the moment. In order to be successful (and be able to meet these requirements in the future), they must be constantly improvement-oriented. One of the few studies on this subject was carried out by Holschbach and Hofmann (2011). One of the directions of further research that they set out was to conduct survey research (their research was based on a case study). Based on the results of surveys, it aims to identify the essential improvement actions that manufacturing companies expect their suppliers to take and check whether their size and management methods affect these expectations.

So far, most of the studies published in the literature on the requirements for suppliers have focused on the buyers’ assessment of the most important parameters such as quality, price and deliveries (Torabi et al. 2015). These criteria are used by clients in the evaluation of suppliers during the initial assessment (selection) and periodic assessment. More and more often, the research results presented in the literature indicate that buyers use multi-criteria assessments of suppliers (Araz and Ozkarahan 2007; Tavana et al. 2021). These multi-criteria assessments, apart from quality, price and deliveries, also include:

- technical and organizational skills of the supplier,
- the width of the offered assortment,
- implementing product / organizational innovations, the flexibility of deliveries,
- pre-sale services (solution design, technical consulting) and.
- after-sale services (installation, technical service, repair and maintenance).
- sustainability (net-zero emissions goals).

It can be noticed that buyers more and more often put expectations of their suppliers in terms of taking improvement actions during the cooperation. There is a research gap in this area in the literature. Therefore, the theoretical considerations and the results of empirical research presented in this article are an attempt to fill this research gap. The purpose of filling this gap was to identify the improvement actions that manufacturing companies expect from their suppliers. It can also be noticed that buyers who have implemented process improvement tools such as international management standards, Lean Management or Toyota Production System have such expectations towards suppliers.

The paper is structured as follows: first, the literature is reviewed and hypotheses developed; second, the data and method are described; third, the results are presented, and the discussion is outlined and fourth, the paper is concluded.

**2. Literature review and research hypotheses**.

### The expectations of companies regarding improvement actions taken by their suppliers

By analyzing the literature and observing the current trends related to the behavior of purchasing enterprises, five groups of variables related to expectations towards suppliers can be distinguished (Li et al. 2021; Tóth et al. 2020). They focus on improving:

1) products,

2) processes,

3) organization management,

4) communication,

5) relationship.

**Improving the quality of products**.

A particularly important expectation towards suppliers is to guarantee the technical quality of products (Negash et al. 2020). This requires strict compliance with the legal requirements relating to safety (included, inter alia, in technical standards, as well as in European Union directives). Guaranteeing technical quality is aimed at ensuring safety (minimizing risks associated with the product) and reliability, the ability to operate without failure during a specified period and conditions of use. For buyers (especially OEMs -Original Equipment Manufacturers), the eco-friendliness of products is also becoming more and more important (Gao et al. 2020). Eco-friendliness is related to reducing the negative impact on the environment of processes such as production, packaging, delivery, use and maintenance of the product, as well as after use (e.g. through recycling or disposal). In order to reduce the negative impact on the environment in the supply chains, companies conduct a life cycle assessment (LCA) (Civancik-Uslu et al. 2019; Jenssen and de Boer 2019; Prosman and Sacchi 2018). These activities are aimed at reducing the amount of materials, energy and waste generated in individual processes. Ensuring the safety and environmental performance of products is of particular importance when purchasing new products (raw materials, materials, parts or infrastructure elements) from new suppliers. For this reason, suppliers to fully meet the expectations of buyers also focus on developing and introducing product innovations to the market. When developing the concept of new (as well as modifying existing) products, more and more companies also require suppliers to implement the eco-design / green design approach (Oroojeni Mohammad Javad et al. 2020; Potter and Graham 2019). With regard to products, this concept consists in analyzing and reducing the negative impact of each product on the environment at all stages of its life cycle (design, production, distribution, installation, use, maintenance, disposal/destruction through dematerialization), or the reuse of materials (recycling ). In order to increase the effectiveness of the safety and environmental performance of products, many buyers on the B2B market undertake joint research and development projects together with their suppliers (Lee et al. 2020; Potter and Graham 2019; Qiu and Yang 2018).

**Process improvement**.

It can also be noticed that expectations towards suppliers increasingly focus on improving the efficiency of delivery (Mohammadivojdan et al. 2022). This efficiency can be increased by improving the timeliness of deliveries and shortening the time of order fulfillment (Prasad h c et al. 2016). The reduction of errors in deliveries and purchase documents is of significant importance for improving the efficiency of order fulfillment (Dupont et al. 2018). These errors are the causes that result in complaint proceedings. Buyers’ expectations also focus on the flexibility of inventory management by suppliers (e.g. through consignment warehouses) or flexibility in the face of changes in delivery orders (the possibility of changing the order as to the date, quantity, sequence or type of product assortment purchased) (Gligor 2020). Timeliness and flexibility of deliveries are of particular importance for buyers who expect suppliers to implement the Just-in-Time concept (Shnaiderman and Ben-Baruch 2016). This is related to the expectations towards suppliers to shorten order fulfillment cycles and thus reduce the costs of operating processes (Jayaram et al. 2010; Ram Kumar et al. 2021). The experiences related to the COVID 19 pandemic show that consignment warehouses and cooperation with suppliers within local cluster structures will be of increasing importance for ensuring the efficiency of purchasing processes.

**Improving organizational management**.

It can also be observed that buyers’ expectations towards suppliers are increasingly focused on implementing not only quality management system but also other tools for improving operational processes. Many enterprises (especially international corporations) expect their suppliers to introduce an EMS (environmental management system) based on the requirements of the ISO 14,001 standard (Arimura et al. 2011; Gurel et al. 2015; Thabit 2021). The requirements of this system focus on improving the environmental impact by:

- reducing the consumption of materials, energy and water,
- elimination of the use of toxic substances, or.
- reducing the emission of gases, noise and electromagnetic waves.

More and more often, suppliers are also obliged to recycle waste, use renewable energy sources, implement electronic communication, and raise the environmental awareness of employees which are the examples of circular economy business model implementation (Awan and Sroufe 2022; Jain et al. 2016; Zhan et al. 2021).

The integration of sustainability in the circular economy models is indicated as an effective supplier action which can be considered to achieve the indicated United Nation’s Sustainable Development Goals or European Green Deal requirements (Awan and Sroufe 2022; Kafel and Nowicki 2022). It can also be observed that purchasing companies expect their suppliers to introduce Toyota Production System elements (such as Kaizen, 5 S, Total Productive Maintenance) and the Lean Management concept. Buyers, wishing to ensure timely and certainty of deliveries, also expect their suppliers to introduce the concept of business continuity (Jacob and Schätzle 2020). The implementation of this concept allows partners in supply chains to become immune to possible disruptions in the continuity of delivery processes (Kaur and Prakash Singh 2021).

**Improving communication**.

Communication processes play an essential role in the development of cooperation between suppliers and buyers (Maestrini et al. 2017). It can be noted that currently, electronic forms of communication between partners are of great importance (Ambrose et al. 2008). An often expected form of communication from suppliers is the exchange of information via the Internet and EDI (Electronic Data Interchange) (Smith et al. 2020). The use of EDI enables the exchange of commercial and financial documents (such as orders, order confirmations, shipping advice, invoices, corrective invoices, etc.) in the form of a standard electronic message, directly between the computer systems of business partners Buyers’ expectations also apply to the use of Enterprise Resource Planning (ERP) solutions by suppliers. Effective communication between partners using the ERP function enables the coordination of the flow of messages between partners in the supply chain. The exchange of information between partners makes it possible to define forecasts of customer demand as well as the actual demand for products. It is possible through close cooperation related to monitoring the state of inventories and production needs of customers (by analyzing the production cycles of individual assortments) (Lin et al. 2011; Ruivo et al. 2020; Syreyshchikova et al. 2020).

**Improving relationships**.

An important condition for cooperation in supply chains is building partnership relations between buyers and suppliers (Patrucco et al. 2021). These relationships are based on mutual trust between partners (Rungsithong and Meyer 2020). This trust can be developed through the use of favorable provisions in contracts between partners. For building partnerships, it is important to enable financial benefits for clients (Wang and Zhang 2021). For this reason, buyers expect guaranteed and turnover bonuses from their suppliers. Increasingly, customers’ expectations also focus on extending the scope of warranty obligations by suppliers, as well as on a wider range of after-sales services (installation, consulting, training, maintenance, upgrading, or collection of the product after decommissioning) (Saccani et al. 2014).

The literature review results indicate that enterprises’ most important expectation is that their suppliers first and foremost improve their products ( Lee et al. 2020; Oroojeni Mohammad Javad et al. 2020; Potter and Graham 2019). All other improvement activities seem to be less critical. It is the basis for the following hypothesis.

H1: *Manufacturing companies expect suppliers, above all, to improve the quality of the delivered products*.

### Factors influencing the company’s expectations regarding their suppliers

The results of many scientific studies have shown that the size of the enterprise is one of the factors that differentiate the practice of their operation (Baumann-Pauly et al. 2013; Sytnik and Kravchenko 2021; Upadhyay et al. 2010). This regularity also applies to aspects related to supply chain management and quality management (Balasubramanian et al. 2020; Wong et al. 2020). This is the basis for the formulation of the second research hypothesis:

H2: *Large enterprises have a higher level of expectations regarding the undertaking of improvement actions by suppliers than medium-sized enterprises*.

Continuous improvement is one of the basic principles of quality management (Kuei and Lu 2013). The management methods applied under this approach will make organizations improve their activities and raise the requirements for their suppliers (Noshad and Awasthi 2015). These methods include, above all, the ISO 9001 Quality Management System, Toyota Production System (TPS), Lean Management, and the ISO 14,001 Environmental Management System (Cress and Fiala 2022; Tomic and Spasojevic Brkic 2019; Yu et al. 2019; Zimon 2017). Therefore, the following hypotheses have been posed:

H3: *Companies with an ISO 9001 QMS have a higher level of expectations regarding the undertaking of improvement actions by their suppliers than companies without an ISO 9001 QMS.*

H4: *Companies using Toyota Production System have a higher level of expectations regarding the undertaking of improvement actions by their suppliers than companies not using TP).*

H5: *Companies applying the Lean Management concept have a higher level of expectations regarding the undertaking of improvement actions by their suppliers than companies not applying the Lean Management concept.*

H6: *Companies with an ISO 14,001 EMS have a higher level of expectations about the environmental performance of their suppliers than companies without an ISO 14,001 EMS.*

## Research methodology

The aim of the study was to define the most important improvement actions that, according to manufacturing companies, their suppliers should take and to check, as well as whether the size of production companies and management tools (ISO 9001, ISO 14,001, TPS, Lean Management) affect these expectations. The study was performed using the Computer Assisted Telephone Interview (CATI) technique. The research covered 118 producers operating on the B2B market (employing over 49 employees) from the automotive, metal and chemical sectors. The study was commissioned to a specialized research agency, which purposefully selected companies registered in the database provided by Bisnode, a leader in the economic information sector in Europe, for many years belonging to the global Dun & Bradstreet network. This database is the largest collection of economic data in Poland (DB CONNECT 2022). As part of the purposeful selection, the following requirements were applied:

- employment of more than 49 employees,
- main activity related to the automotive, chemical or metal industries.

The surveyed companies assigned one of two answers to the indicated expectations towards suppliers regarding ensuring and improving the quality of products and processes: Action is significant, or Action does not matter. If the factor was significant for the respondent, it additionally indicated appropriate ranks on a scale from 5 (the most important criterion) to 1 (the least important). Consequently, each answer was asses on a 6-point scale, as in Table 1.

**Table 1:** The scale of grades used in the study

Scale	0	1	2	3	4	5
Answer	Not Important	Important				
		Less Important	Slightly Important	Moderately Important	Important	Most Important

Source: own study

The respondents defined their expectations in five main categories to which individual requirements were assigned. The main categories of expectations, along with detailed actions resulting from the literature review, are shown in Table 2.

**Table 2:** The main categories of improvement expectations with detailed actions

Z1. Product Improvement Improve the quality of products Improve the environmental performance of products Introduce product innovations
**Z2. Process Improvement** Shorten the order fulfillment time Improve the timeliness of deliveries Reduce delivery errors Reduce errors in purchasing documents Manage your inventory flexibly Increase flexibility in the face of changes to orders Reduce the costs of operational processes
**Z3. Organizational Management Improvement** Introduce the concept of ensuring business continuity Introduce systemic environmental management according to ISO 14,001 Introduce the concept of Lean Management Introduce the elements of the Toyota Production System (Kaizen, 5 S, TPM)
**Z4. Improving communication** Improve communication through electronic contact via the Internet or the EDI system Use an ERP tool
**Z5. Improving relationships** Use other favorable provisions in contracts, depending on the ongoing cooperation Extend the scope of warranty obligations Apply a system of guaranteed and spinning trade bonuses (bonuses) Extend the range of after-sales services

Source: own study

The five main categories of expectations, presented in Table 2, were determined based on the median of the obtained results for all subcategories included in the form. Those categories were measured of internal consistency reliability by calculating the Cronbach’s Alpha. The obtained results ranged from 0.55 for the Z4 category to 0.81 for the Z2 category. Considering the number of questions for individual variables can be indicated that individual categories are internally consistent. (Tavakol and Dennick 2011). The consistency level for the total scale was 0,91, which can be described as an Excellent result according to the rule provided by George and Mallery (George and Mallery 2002). In order to verify the research hypothesis, the non-parametric Mann-Whitney U test was used with the *p* value lower than 0,05. As stated by many researchers, it is one of the most powerful and common used non-parametric tests (Landers 1981).

## Results

Almost all of the business entities participating in the study had implemented a quality management system based on the requirements of the international ISO 9001 standard. Only in two enterprises, this system was not certified. 52.5% of the surveyed companies had implemented environmental management systems based on the international ISO 14,001 standard requirements. Implementation of Lean management and usage of TPS was declared respectively by 16,9% and 28,0% of studied organizations. Almost half of the respondents (44.9%) were enterprises with foreign capital. More than half of the surveyed population were organizations employing over 250 employees − 55.9%. The remaining organizations employed from 50 to 250 employees (44.1%). The main types of business activity are the automotive industry (40.6%), chemical industry (34.8%) and metal industry 24.6%.

**Fig. 1:**
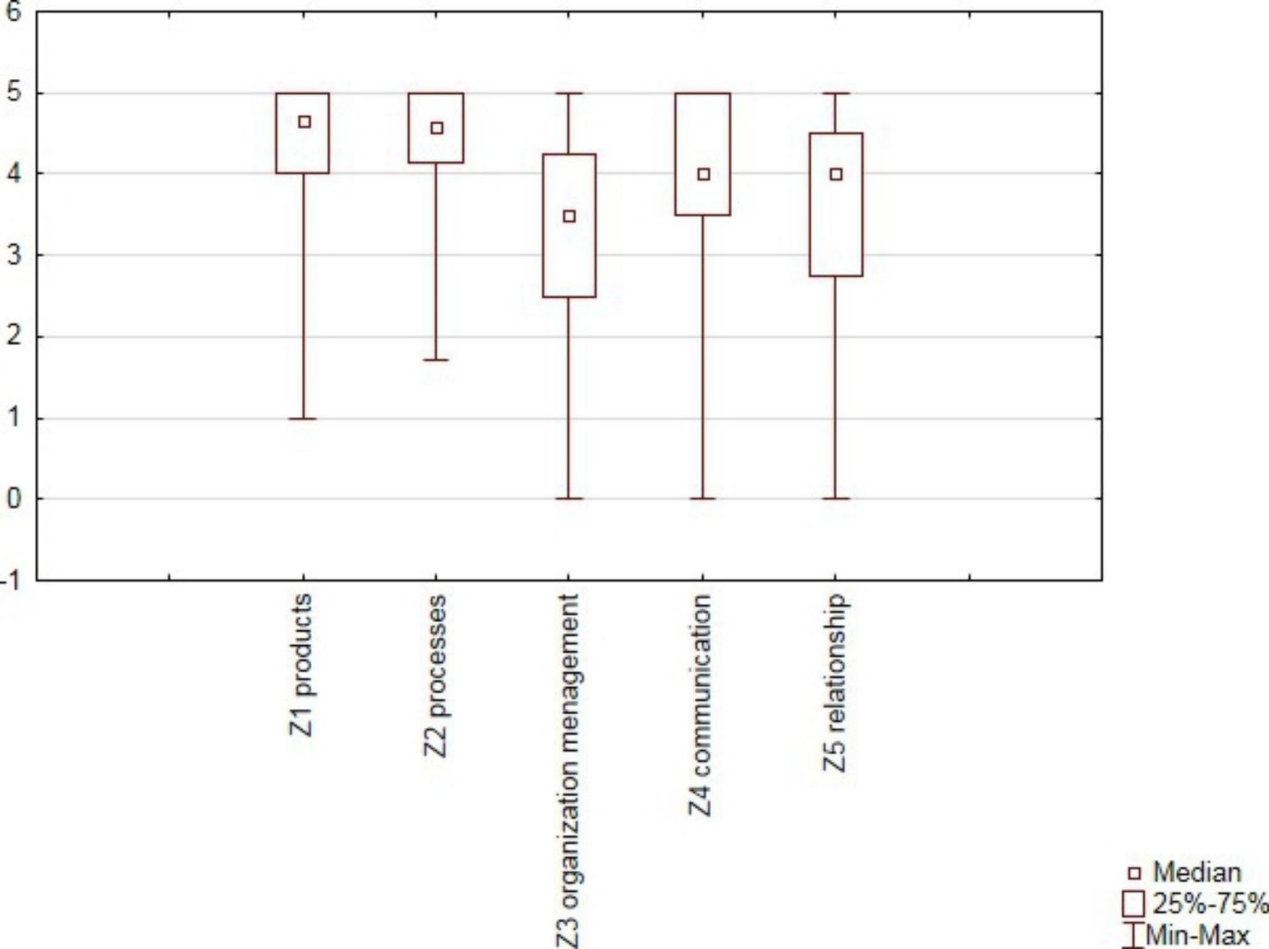
Distribution of results for the five categories of manufacturers’ expectations

Source: own study.

The survey results show high expectations of manufacturing companies in relation to their suppliers in all five categories. The most important for the studied organizations were the first and second categories of expectations, which concerned the improvement of products and the requirements related to the improvement of processes. For these categories, the median was above 4; additionally, none of the analyzed organizations indicated that the requirements included in individual variable categories were *not valid*. A detailed distribution for individual categories is presented in Fig. 1.

H1: *Manufacturing companies expect suppliers, above all, to improve the quality of the delivered products.*

The verification of the H1 hypothesis was carried out using the Mann-Whitney U test, where the responses for group Z1 (i.e. product quality improvement) were compared with the other groups specified in the study (see Table 3).

**Table 3:** Compare the differences between the types of expectations - the Mann-Whitney U test

Types of expectations	U	Z	Significance level - p	Statistically significant difference
Z1 vs. Z2	6711,500	0,476732	p = 0,6287	No
Z1 vs. Z3	3353,500	6,880199	p < 0,0001	Yes
Z1 vs. Z4	5554,500	2,683049	p = 0,0073	Yes
Z1 vs. Z5	4273,500	5,125824	p < 0,0001	Yes

Source: own study

The analysis results do not allow for the accept the H1 hypothesis. Although the scores for the Z1 category are significantly higher than for Z3, Z4 and Z5 categories, no statistically significant difference was found between Z1 and Z2.

However, hypothesis 1 can be slightly reformulated. It can be assumed that manufacturing companies expect suppliers to improve the quality of the delivered products and business processes (H1a hypothesis). The results of the U-Man Whitney test after combining groups Z1 and Z2 and comparisons with the other groups are presented in Table 4.

**Table 4:** Comparison of differences between Z1 + Z2 and other types of expectations - Mann-Whitney U test

Category	U	Z	Significance level - p	Statistically significant difference
Z1 + Z2 vs. Z3	3098,500	7,366465	p < 0,001	Yes
Z1 + Z2 vs. Z4	5605,000	2,586749	p = 0,009	Yes
Z1 + Z2 vs. Z5	4166,500	5,329866	p < 0,001	Yes

Source: own study

The conducted analysis clearly showed a basis for accepting the H1a hypothesis.

H2: *Large enterprises have higher expectations regarding the undertaking of improvement actions by suppliers than medium-sized enterprises.*

The average rating for the improvement requirements imposed on suppliers by large enterprises was 4.30, while for medium-sized organizations, the value was 3.95. The performed U-Mana Whitney test showed that the difference between these values ​​is statistically significant (p = 0.030). On this basis, hypothesis 2 was accepted. Large enterprises have higher expectations regarding the undertaking of improvement actions by suppliers than medium-sized enterprises. This finding is not surprising as it is consistent with the results of many studies which have shown that large enterprises are characterized by a higher level of innovation (Noori et al. 2017), productivity (Kim and Ro 2017), customer satisfaction (Heiens et al. 2019) and, above all, advances in quality management than smaller organizations. Erginel (2010) showed, for example, that large enterprises cope better with the implementation of the principles of quality management, especially in the field of leadership, employee involvement, continuous improvement and decision based on facts. On the other hand, Eriksson (2016), analyzing the differences among SMEs and large organizations regarding the outcome of quality management practices, found that large organizations are ahead of small and medium enterprises in the race for quality progress. So it is no surprise that they have higher expectations of their suppliers than smaller companies.

A detailed analysis of the collected information showed statistically significant differences in the level of requirements concerning one area, mainly: relationship improvement (see Table 5).

**Table 5:** Company size versus expectations regarding improvement actions

Main improvement categories	Expectations – median for the group		Statistically significant difference
	Big companies	Medium companies	
products	4,67	4,50	not confirmed
processes	4,57	4,57	not confirmed
organization management	3,88	3,38	not confirmed
communication	4,00	4,00	not confirmed
relationship	4,00	3,63	**confirmed**

The lack of differentiation in the scope of expectations regarding the improvement of organizational management may be considered a kind of surprise.

H3: *Companies with an ISO 9001 QMS have a higher level of expectations regarding the undertaking of improvement actions by their suppliers than companies without an ISO 9001 QMS.*

Due to the significant number of organizations with an implemented and certified QMS compliant with the requirements of ISO 9001, the verification of the H3 hypothesis was not possible. In the analyzed sample of 118 organizations, only two manufacturers indicated the lack of such a system.

H4: *Companies using TPS (Kaizen, 5 S, TPM) have a higher level of expectations regarding the taking of actions related to process improvement by their suppliers than companies not using TPS (Kaizen 5 S, TPM).*

The median assessment of expectations related to undertaking improvement actions by their suppliers by producers using TPS (Kaizen, 5 S, TPM) was 4.40, while in the case of enterprises not using these tools, it was 4.00. The U-Man Whitney test showed that the difference between these values ​​is statistically significant (p = 0.009). On this basis, hypothesis 4 was accepted (see Table 6).

**Table 6:** TPS versus expectations regarding improvement actions

Main improvement categories	Expectations – median for the group		Statistically significant difference
	With TPS	Without TPS	
products	4,67	4,67	not confirmed
processes	4,71	4,57	**confirmed**
organization management	4,00	3,25	**confirmed**
communication	4,50	4,00	**confirmed**
relationship	4,00	3,75	not confirmed

H5: *Companies applying the Lean Management concept have a higher level of expectations regarding the undertaking of improvement actions by their suppliers than companies not applying the Lean Management concept.*

The median rating of expectations related to undertaking improvement actions by their suppliers by producers applying the Lean Management concept was 4.32, while in the case of enterprises not applying this concept, it was 4.05. The U-Man Whitney test showed that the difference between these values ​​is not statistically significant (p = 0.119). On this basis, hypothesis 5 was not accepted. Efficient implementation of Lean Management projects by partners in supply chains undoubtedly contributes to improving the timeliness of deliveries (shortening production cycles) and reducing the costs of operating processes (Hofer et al. 2021; Sunil Kumar et al. 2018). Enterprises that have implemented Lean Management have a higher level of expectations from suppliers regarding the undertaking of improvement actions within the organization management category than organizations that have not implemented Lean Management (see Table 7).

**Table 7:** Lean Management versus expectations regarding improvement actions

Main improvement categories	Expectations – median for the group		Statistically significant difference
	With Lean Management	Without Lean Management	
products	4,33	4,67	not confirmed
processes	4,71	4,57	**confirmed**
organization management	4,13	3,50	**confirmed**
communication	4,25	4,00	**confirmed**
relationship	3,88	4,00	not confirmed

The lack of significant differences in product quality requirements can be explained by a very high level of expectations in the entire industry, confirmed by the research conducted. The decision to choose a TPS or LM is motivated by internal or external premisses. However, no matter what choice is made, the quality requirements of the supplier’s product will always be high.

H6: *Companies with an ISO 14,001 EMS have a higher level of expectations about the environmental performance of their suppliers than companies without an ISO 14,001 EMS.*

Environmental performance of products was one of the subcategories of the Product Improvement category of expectations (see Table 2). That subcategory was used to verify the H6 hypothesis. The median rating of related requirements *environmental performance of products* by the group of organizations with ISO 14,001 EMS was 5.

For the group organizations without such a system, the median was 4. The U-Man Whitney test showed that the difference between these groups of organizations is not statistically significant (p = 0.217). There is insufficient evidence to support the H6 hypothesis. There is also no significant difference for the products improvement category in which *environmental performance of products* is a subcategory. A detailed analysis of the results indicates that differences in the level of requirements concern only one category, which is organization management (see Table 8).

**Table 8:** ISO 14,001 versus expectations regarding improvement actions

Main improvement categories	Expectations – median for the group		Statistically significant difference
	With ISO 14,001	Without ISO 14,001	
products	4,67	4,67	not confirmed
processes	4,71	4,57	**confirmed**
organization management	3,88	3,38	**confirmed**
communication	4,00	4,00	**confirmed**
relationship	4,00	3,75	not confirmed

**Results discussion**.

The verification oh H1a hypothesis means that manufacturing companies expect their suppliers to improve the quality of the delivered products and business processes. The obtained results confirm the belief presented in the literature on the subject that it is vital for enterprises operating on the B2B market that their suppliers provide an appropriate method of management, communication and relations (see Kian Chong et al. 2011; Kaski et al. 2017; Sales-Vivó et al. 2021), at the same time reminded that the most important thing is to improve products and business processes.

The literature emphasizes that SMEs are “forced/inspired” by their clients to introduce new management methods/systems. On the other hand, large enterprises decide to implement new solutions for other reasons, such as the desire to reduce costs or corporate policy (Sun and Cheng 2002). It could therefore be assumed that large enterprises have greater expectations regarding the improvement of organization management by their suppliers. However, the results of our research did not confirm this view. The results of the research conducted by (Sun and Cheng 2002) may help understand this situation. On their basis, it can be assumed that it is the position in the supply chain, not the company’s size, that has a significant impact on the expectations regarding the implementation of new management methods/systems by suppliers. It is possible that a more important factor differentiating the examined characteristics is the level of knowledge resources obtained by manufacturing companies rather than the size of the manufacturer. That factor, according to Awan et al. (2020) plays is an important intervening variable in organizational sustainability of suppliers.

That result concerning the popularity of the QMS (hypothesis H3) confirms the observations of other researchers. In the automotive industry, OEMs are interested in certifying all their sub-suppliers, including tier 1 and tier 2, for compliance with the requirements, e.g. IATF 16,949, which is based on the prior fulfillment of the requirements of the ISO 9001 standard (Laskurain-Iturbe et al. 2021; Singh 2014). There is in the literature a line of studies that point out the resignation from QMS certification, primarily by bigger organizations from Europe (Cândido et al. 2021; Kafel and Nowicki 2014; Simon and Kafel 2018). It is a new trend, but it is not the case in a studied industry where ISO 9001 certification is still a “must-have” issue. This is in line with Ferreira et al. (2021) studies where external variable such as supply chain relationships or country of origin can play an important role in the certification withdrawal.

Another interesting part of the results in the TPS influence on the supplier expectations. Companies that have implemented the Toyota Production System have higher expectations regarding the undertaking of improvement actions (concerning processes and management methods) by suppliers than organizations that have not implemented these tools. The implementation by partners operating in the supply chains of TPS tools undoubtedly improves communication and flexibility of cooperation between them, as well as entitles the delivery processes (especially custom-made products and produced in short series) (Paladugu and Grau 2020; Ram Kumar et al. 2021).

Presented results related to the ISO 14,001 EMS do not seem surprising. The lack of significant differences in the level of expectations regarding products results from the generally very high level of expectations by all surveyed organizations. Considering the green product and process innovations expectation, it is possible that internal competencies and the role of buyers in knowledge transfer are more important than just certification of EMS (Awan et al. 2021). EMS focuses more on mitigating negative environmental effects caused by processes than on final products from the management perspective (ISO 2015). This may explain the higher expectations regarding the organization management significantly (see Table 8). Continuous improvement is the basis for the functioning of all management meta-standards, including the EMS system compliant with ISO 14,001 (Heras-Saizarbitoria and Boiral 2013). Higher expectations of organizations with EMSs towards suppliers without such a system may result from the willingness to impose on business partners a similar management model that they have introduced themselves. Similar conclusions can be made in terms of communication expectations. According to the Fonseca and Domingues research (2018), internal and external communication is an important element of the activities of organizations that implement EMS.

**Perceptible trends related to the supplier development programs**.

More and more purchasing companies not only define their expectations towards suppliers in terms of taking improvement actions but also try to help them by offering development programs (Jafarian et al. 2021). These programs are based on offering training and consultancy in the field of product quality assurance, implementation of system tools (in terms of improving environmental impact and improving process safety) (Bai and Satir 2020; Fan et al. 2021). More and more often, supplier development programs focus on the implementation of operational improvement tools, such as elements of the Toyota Production System or Lean Management (Arlinghaus and Knizkov 2020). Through the transfer of knowledge, it is possible to implement joint projects aimed at the development of product innovations, solving technical and organizational problems, which allows for greater efficiency and effectiveness in improving the processes carried out in the supply chain (Saghiri and Wilding 2021). The implementation of the Toyota Production System and Lean Management allows to shorten the cycles of implementation time and reduce the costs associated with the use of material resources by reducing losses, unnecessary operations or over-exploitation of the infrastructure (Nagati and Rebolledo 2013; Pradhan and Routroy 2018). More and more often, supplier development programs focus on the implementation of CSR and sustainability concepts that assume achieving goals in the form of target indicators related to environmental protection (such as reducing the consumption of harmful substances, carbon dioxide emissions), improving product safety (reducing the number of manufacturing defects, customer complaints) and processes (reducing the risk of accidents or emergencies) (Akman 2015; Awasthi and Kannan 2016; Dou et al. 2014).

**Conclusions**.

This study investigated the expectations of manufacturing companies regarding improvement actions taken by their suppliers. The key contribution to the existing literature of this study is to deepen understanding of the expectations of manufacturing companies towards suppliers which focus on improving products, processes, implemented management tools, mutual communication and building beneficial relationships. Previously conducted research focused on supplier evaluation by purchasing companies. This study contributes to the theory by filling the existing gap between important improvement actions that manufacturing companies expect their suppliers to take in the future and well know requirements that are used in supplier assessment processes. Evaluation of suppliers is carried out by buyers using the criteria of initial assessment and periodic assessment. The results of the research presented in the article show that manufacturing companies expect their suppliers to develop by improving their activities. The results of these studies indicate that this development should be carried out by suppliers through the improvement of products, processes, implemented management tools, mutual communication and building of beneficial relationships. In relation in supply chains, the improvement of products and processes by providers is of particular importance for buyers. The research results indicate that when improving products, suppliers should focus on improving safety and environmental performance as well as increasing innovation. In turn, by improving the processes, suppliers should shorten the time of order fulfilment, reduce errors and reduce operating costs. The results of the research also showed that the expectations of producers towards suppliers also apply to the implementation of organizational improvement tools (such as the environmental management system, Toyota Production System, or Lean Management). When analyzing global trends, it can be concluded that customer expectations towards suppliers in the future will focus to a greater extent on the implementation of these tools. The research results also show that expectations towards suppliers also concern the improvement of the effectiveness of mutual communication processes and building partnership relations based on mutual benefits. Meeting these expectations formulated by buyers undoubtedly allows improving the potential of supplier resources and processes implemented by partners in supply chains. Product and process improvement is very important for buyers. Manufacturing companies expect their suppliers to improve in the future the quality of the delivered products and business processes more than improvement in such areas as communication, organization management and relationships. Large enterprises have higher expectations regarding the undertaking of improvement actions by suppliers than medium-sized enterprises. Moreover, companies that have implemented the Toyota Production System have a higher level of expectations regarding the undertaking of improvement actions, mainly in the area of processes, management methods and communication, by suppliers than manufacturing organizations that have not implemented TPS. Manufacturing companies that have implemented Lean Management have a higher level of expectations from their suppliers, regarding the undertaking of improvement actions within the organization management category than organizations that have not implemented Lean Management. According to the results of the study, there are higher expectations of organizations with EMSs towards improvement in the category of organization management. Generalizing large enterprises that have implemented organizational improvement tools (such as the environmental management system, Toyota Production System or Lean Management) pay particular attention to the improvement by their suppliers as a continuation of cooperation requirement. When analyzing global trends, it can be concluded that customer expectations towards suppliers in the future will focus to a greater extent on the implementation of these tools. Buying companies that have implemented these tools recognize that their use contributes to the improvement of products and processes in supply chains.

From the managerial point of view, the considerations presented in the article show that the requirements for suppliers are not limited to the criteria of initial and periodic assessment. These requirements increasingly focus on the necessity to take actions aimed at continuous improvement by suppliers. Assessment of compliance with these requirements is more and more often in the form of periodic reporting of results by suppliers and their verification through audits. Such observations broaden the literature discussion of the analyzed research problem. The results of the presented empirical research indicate areas of activities that, in the opinion of buyers, should be improved by suppliers. Indication of these improvement areas may constitute a recommendation for enterprises operating in supply chains. In practice, recommending improvement actions to providers should increase the efficiency of supply chains.

Based on the findings of the presented study, development trends and implications for managers responsible for purchasing processes and relationships with suppliers can be determined. These implications assume that the effective fulfillment of suppliers’ expectations of purchasing enterprises undoubtedly requires close cooperation between the partners. This collaboration should focus on helping buyers to help suppliers meet improvement expectations. Managers responsible for purchasing processes and supplier relationships should be involved in this assistance. This assistance is based on the joint implementation of projects focusing on improving products and processes. In joint project implementation, purchasing companies could offer to consult and train suppliers in tools enabling the implementation of tools for improving products and processes in supply chains. Effective implementation of such projects allows buyers and suppliers to improve communication processes and build mutual relationships based on win-win principles. In practice, it can be observed that more and more international concerns are trying to implement such projects by offering supplier development programs. Such projects often focus on the joint implementation of research and development works on new products and process improvement through the implementation of the Toyota Production System or the Lean Management concept. Such practical cooperation between buyers and providers contributes to shortening order fulfillment cycles and improving the efficiency of processes by reducing costs in the supply chain.

The findings of the study are also constrained by limitations and open some paths for future research. The limitation of the study is that the research was carried out in enterprises operating on the Polish market. However, almost half of the surveyed organizations were enterprises with foreign capital, which are very active in the business on international markets. Future research studies may ought to investigate the buyers’ expectations towards suppliers in terms of implementing the concept of sustainability, as well as the concept of business continuity management (Coşkun et al. 2022; Tseng et al. 2022). It can be observed that the role of the concept of sustainability in relation with suppliers is constantly increasing (Fan et al. 2021; Hosseini et al. 2022). The expectations of buyers towards suppliers will focus more on reducing the consumption of raw materials and energy, as well as on reducing gas emissions (to reduce the carbon footprint) (Hashmi et al. 2021). On the other hand, expectations towards suppliers regarding the implementation of the business continuity management concept should ensure the resilience of supply chains to possible disruptions (Hosseini et al. 2019; Shin and Park 2021).

**Declarations**.

### Financial interests

The authors have no competing interests to declare that are relevant to the content of this article.

**Funding**:

The publication has been co-financed by the subsidy granted to the University of Lodz.

The publication has been co-financed by the subsidy granted to the Cracow University of Economics.

The project is co-financed by the Ministry of Science and Higher Education of Poland under the program “Regional Initiative of Excellence” 2019–2022, project number 015 / RID / 2018/19, total funding amount 10 721 040.00 PLN.

## References

[CR1] Afraz MF, Bhatti SH, Ferraris A, Couturier J (2021). The impact of supply chain innovation on competitive advantage in the construction industry: Evidence from a moderated multi-mediation model. Technol Forecast Soc Change.

[CR2] Akman G (2015). Evaluating suppliers to include green supplier development programs via fuzzy c-means and VIKOR methods. Comput Ind Eng.

[CR3] Ambrose E, Marshall D, Fynes B, Lynch D (2008). Communication media selection in buyer-supplier relationships. Int J Oper Prod Manag.

[CR4] André Mendes Primo M, Dooley K, Johnny Rungtusanatham M (2007). Manufacturing firm reaction to supplier failure and recovery. Int J Oper Prod Manag.

[CR5] Araz C, Ozkarahan I (2007). Supplier evaluation and management system for strategic sourcing based on a new multicriteria sorting procedure. Int J Prod Econ.

[CR6] Arimura TH, Darnall N, Katayama H (2011). Is ISO 14001 a gateway to more advanced voluntary action? The case of green supply chain management. J Environ Econ Manage.

[CR7] Arlinghaus JC, Knizkov S (2020). Lean Maintenance and Repair Implementation - A Cross-Case Study of Seven Automotive Service Suppliers. Procedia CIRP.

[CR8] Awan U, Arnold MG, Gölgeci I (2021). Enhancing green product and process innovation: Towards an integrative framework of knowledge acquisition and environmental investment. Bus Strateg Environ.

[CR9] Awan U, Khattak A, Rabbani S, Dhir A (2020). Buyer-Driven Knowledge Transfer Activities to Enhance Organizational Sustainability of Suppliers. Sustainability.

[CR10] Awan U, Sroufe R (2022). Sustainability in the Circular Economy: Insights and Dynamics of Designing Circular Business Models. Appl Sci.

[CR11] Awasthi A, Kannan G (2016). Green supplier development program selection using NGT and VIKOR under fuzzy environment. Comput Ind Eng.

[CR12] Azadi M, Jafarian M, Farzipoor Saen R, Mirhedayatian SM (2015). A new fuzzy DEA model for evaluation of efficiency and effectiveness of suppliers in sustainable supply chain management context. Comput Oper Res.

[CR13] Bai C, Satir A (2020). Barriers for green supplier development programs in manufacturing industry. Resour Conserv Recycl.

[CR14] Balasubramanian S, Shukla V, Chanchaichujit J (2020). Firm size implications for environmental sustainability of supply chains: evidence from the UAE. Manag Environ Qual An Int J.

[CR15] Basu A, Jain T, Hazra J (2018). Supplier selection under production learning and process improvements. Int J Prod Econ.

[CR16] Baumann-Pauly D, Wickert C, Spence LJ, Scherer AG (2013). Organizing Corporate Social Responsibility in Small and Large Firms: Size Matters. J Bus Ethics.

[CR17] Cândido CJF, Coelho LMS, Peixinho RMT (2021). Why firms lose their ISO 9001 certification: Evidence from Portugal. Total Qual Manag Bus Excell.

[CR18] Chen H, Jeter D (2008). The Role of Auditing in Buyer-Supplier Relations. J Contemp Account Econ.

[CR19] Civancik-Uslu D, Puig R, Voigt S (2019). Improving the production chain with LCA and eco-design: application to cosmetic packaging. Resour Conserv Recycl.

[CR20] Coşkun SS, Kumru M, Kan NM (2022). An integrated framework for sustainable supplier development through supplier evaluation based on sustainability indicators. J Clean Prod.

[CR21] Cress P, Fiala T (2022). Adapting the Toyota Production System in Plastic Surgery Practices to Improve Practice Management and the User Experience. Aesthetic Surg J.

[CR117] DB CONNECT. 2022.https://www.all-for-one.pl/pl/poradnik/db-connect-integracja-sap-z-baza-bisnode-db/

[CR22] Dou Y, Zhu Q, Sarkis J (2014). Evaluating green supplier development programs with a grey-analytical network process-based methodology. Eur J Oper Res.

[CR23] Dupont L, Bernard C, Hamdi F, Masmoudi F (2018). Supplier selection under risk of delivery failure: a decision-support model considering managers’ risk sensitivity. Int J Prod Res.

[CR24] Eid R, Trueman M, Moneim Ahmed A (2002). A cross-industry review of B2B critical success factors. Internet Res.

[CR25] Erginel N (2010). No TitleAre TQM Principles Implemented by Large Companies and Smes Similar in Turkey?. Anadolu Univ Sci Technol - A Appl Sci Eng.

[CR26] Eriksson H (2016). Outcome of quality management practices. Int J Qual Reliab Manag.

[CR27] Fan D, Xiao C, Zhang X, Guo Y (2021). Gaining customer satisfaction through sustainable supplier development: The role of firm reputation and marketing communication. Transp Res Part E Logist Transp Rev.

[CR28] Ferreira LMFR, Cândido CJF (2021). Factors influencing firm propensity for ISO 9001 withdrawal: Evidence on decertification tendency and antecedents. Int J Prod Econ.

[CR29] Fonseca L, Domingues J (2018). Exploratory Research of ISO 14001:2015 Transition among Portuguese Organizations. Sustainability.

[CR30] Gao H, Ju Y, Santibanez Gonzalez EDR, Zhang W (2020). Green supplier selection in electronics manufacturing: An approach based on consensus decision making. J Clean Prod.

[CR31] Gelderman CJ, van Hal L, Lambrechts W, Schijns J (2021). The impact of buying power on corporate sustainability - The mediating role of suppliers’ traceability data. Clean Environ Syst.

[CR32] George D, Mallery P (2002) SPSS for Windows Step by Step: A Simple Guide and Reference, 11.0 Update. Allyn & Bacon; 4th edition (August 19, 2002), Boston

[CR33] Gligor DD (2020). Birds of a feather: The impact of race on the supplier selection and evaluation process. Int J Prod Econ.

[CR34] Gurel O, Acar AZ, Onden I, Gumus I (2015). Determinants of the Green Supplier Selection. Procedia - Soc Behav Sci.

[CR35] Hashmi N, Jalil SA, Javaid S (2021). Carbon footprint based multi-objective supplier selection problem with uncertain parameters and fuzzy linguistic preferences. Sustain Oper Comput.

[CR36] Heras-Saizarbitoria I, Boiral O (2013). ISO 9001 and ISO 14001: Towards a Research Agenda on Management System Standards*. Int J Manag Rev.

[CR37] Hofer C, Barker J, Eroglu C (2021). Interorganizational imitation in supply chain relationships: The case of inventory leanness. Int J Prod Econ.

[CR38] Holschbach E, Hofmann E (2011). Exploring quality management for business services from a buyer’s perspective using multiple case study evidence. Int J Oper Prod Manag.

[CR39] Hosseini S, Morshedlou N, Ivanov D (2019). Resilient supplier selection and optimal order allocation under disruption risks. Int J Prod Econ.

[CR40] Hosseini ZS, Flapper SD, Pirayesh M (2022). Sustainable supplier selection and order allocation under demand, supplier availability and supplier grading uncertainties. Comput Ind Eng.

[CR41] Islam ASMT (2019). End of the day, who is benefited by Lean Manufacturing? A dilemma of communication and pricing in buyer-supplier relationship. Manuf Lett.

[CR42] ISO (2015) ISO 14001:2015 Environmental management systems. Requirements with guidance for use

[CR43] Jacob F, Schätzle S (2020). Will a supplier’s origin make a difference to its business customers?. Ind Mark Manag.

[CR44] Jafarian M, Lotfi MM, Pishvaee MS (2021). Supplier switching versus supplier development under risk: A mathematical modelling approach. Comput Ind Eng.

[CR45] Jain V, Kumar S, Kumar A, Chandra C (2016). An integrated buyer initiated decision-making process for green supplier selection. J Manuf Syst.

[CR46] Jayaram J, Das A, Nicolae M (2010). Looking beyond the obvious: Unraveling the Toyota production system. Int J Prod Econ.

[CR47] Jenssen MM, de Boer L (2019). Implementing life cycle assessment in green supplier selection: A systematic review and conceptual model. J Clean Prod.

[CR48] Kafel P, Nowicki P(2014) Functioning of environmental and quality management systems after resignation of management standard certification: Case study of a polish organizations.Int J Qual Res8

[CR49] Kafel P, Nowicki P, Wojnarowska M, Ćwiklicki M, Ingrao C (2022). Significance and adjustment of environmental certification schemes in the Circular Economy. Sustainable Products in the Circular Economy.

[CR50] Kaski TA, Hautamaki P, Pullins EB, Kock H (2017). Buyer versus salesperson expectations for an initial B2B sales meeting. J Bus Ind Mark.

[CR51] Kaur H, Prakash Singh S (2021). Multi-stage hybrid model for supplier selection and order allocation considering disruption risks and disruptive technologies. Int J Prod Econ.

[CR52] Kian Chong W, Shafaghi M, Leing Tan B (2011). Development of a business-to‐business critical success factors (B2B CSFs) framework for Chinese SMEs. Mark Intell Plan.

[CR53] Kim JW, Ro Y-J (2017). The productivity spillover between SMEs and large firms in Korea. Ind Corp Chang.

[CR54] Kuei C, Lu MH (2013). Integrating quality management principles into sustainability management. Total Qual Manag Bus Excell.

[CR55] Landers J (1981) Quantification in history, topic 4: Hypothesis testing II-differing central tendency. Oxford All Souls Coll

[CR56] Laskurain-Iturbe I, Arana-Landín G, Heras-Saizarbitoria I, Boiral O (2021). How does IATF 16949 add value to ISO 9001? An empirical study. Total Qual Manag Bus Excell.

[CR57] Lee NC-A, Wang ETG, Grover V (2020). IOS drivers of manufacturer-supplier flexibility and manufacturer agility. J Strateg Inf Syst.

[CR58] Li S, Peng G, Xing F (2021). Value co-creation in industrial AI: The interactive role of B2B supplier, customer and technology provider. Ind Mark Manag.

[CR59] Lin C-T, Chen C-B, Ting Y-C (2011). An ERP model for supplier selection in electronics industry. Expert Syst Appl.

[CR60] Liou JJH, Chang M-H, Lo H-W, Hsu M-H (2021). Application of an MCDM model with data mining techniques for green supplier evaluation and selection. Appl Soft Comput.

[CR61] Liou JJH, Chuang Y-C, Zavadskas EK, Tzeng G-H (2019). Data-driven hybrid multiple attribute decision-making model for green supplier evaluation and performance improvement. J Clean Prod.

[CR62] Lou Z, Ye A, Mao J, Zhang C (2022). Supplier selection, control mechanisms, and firm innovation: Configuration analysis based on fsQCA. J Bus Res.

[CR63] Maestrini V, Luzzini D, Maccarrone P, Caniato F (2017). Supply chain performance measurement systems: A systematic review and research agenda. Int J Prod Econ.

[CR64] Maestrini V, Martinez V, Neely A (2018). The relationship regulator: a buyer-supplier collaborative performance measurement system. Int J Oper Prod Manag.

[CR65] Mittal V, Han K, Lee J-Y, Sridhar S (2021). Improving Business-to-Business Customer Satisfaction Programs: Assessment of Asymmetry, Heterogeneity, and Financial Impact. J Mark Res.

[CR66] Mohammadivojdan R, Merzifonluoglu Y, Geunes J (2022). Procurement portfolio planning for a newsvendor with supplier delivery uncertainty. Eur J Oper Res.

[CR67] Nagati H, Rebolledo C (2013). Supplier development efforts: The suppliers’ point of view. Ind Mark Manag.

[CR68] Negash YT, Kartika J, Tseng M-L, Tan K (2020). A novel approach to measure product quality in sustainable supplier selection. J Clean Prod.

[CR69] Nikoofal ME, Gümüş M (2020). Value of audit for supply chains with hidden action and information. Eur J Oper Res.

[CR70] Nikookar E, Varsei M, Wieland A (2021). Gaining from disorder: Making the case for antifragility in purchasing and supply chain management. J Purch Supply Manag.

[CR71] Noori J, Bagheri Nasrabadi M, Yazdi N, Babakhan AR (2017). Innovative performance of Iranian knowledge-based firms: Large firms or SMEs?. Technol Forecast Soc Change.

[CR72] Noshad K, Awasthi A (2015). Supplier quality development: A review of literature and industry practices. Int J Prod Res.

[CR73] Oroojeni Mohammad Javad M, Darvishi M, Oroojeni Mohammad Javad A (2020). Green supplier selection for the steel industry using BWM and fuzzy TOPSIS: A case study of Khouzestan steel company. Sustain Futur.

[CR74] Paladugu BSK, Grau D (2020) Toyota Production System – Monitoring Construction Work Progress With Lean Principles. Encyclopedia of Renewable and Sustainable Materials. Elsevier, pp 560–565

[CR75] Patrucco AS, Moretto A, Knight L (2021). Does relationship control hinder relationship commitment? The role of supplier performance measurement systems in construction infrastructure projects. Int J Prod Econ.

[CR76] Potter A, Graham S (2019). Supplier involvement in eco-innovation: The co-development of electric, hybrid and fuel cell technologies within the Japanese automotive industry. J Clean Prod.

[CR77] Pradhan SK, Routroy S (2018). Improving supply chain performance by Supplier Development program through enhanced visibility. Mater Today Proc.

[CR78] Prasad hc, Kamath S, Barkur G, Naik G (2016). Does supplier evaluation impact process improvement?. J Ind Eng Manag.

[CR79] Prosman EJ, Sacchi R (2018). New environmental supplier selection criteria for circular supply chains: Lessons from a consequential LCA study on waste recovery. J Clean Prod.

[CR80] Qiu T, Yang Y (2018). Knowledge spillovers through quality control requirements on innovation development of global suppliers: The firm size effects. Ind Mark Manag.

[CR81] Ram Kumar S, Nimesh Nathan V, Mohammed Ashique SI (2021). Productivity enhancement and cycle time reduction in toyota production system through jishuken activity – Case study. Mater Today Proc.

[CR82] Rashid Khan H, Awan U, Zaman K et al (2021) Assessing Hybrid Solar-Wind Potential for Industrial Decarbonization Strategies: Global Shift to Green Development. Energies 14:7620. 10.3390/en14227620

[CR83] Ruivo P, Johansson B, Sarker S, Oliveira T (2020). The relationship between ERP capabilities, use, and value. Comput Ind.

[CR84] Rungsithong R, Meyer KE (2020). Trust and knowledge sharing in context: A study of international buyer-supplier relationships in Thailand. Ind Mark Manag.

[CR85] Saccani N, Visintin F, Rapaccini M (2014). Investigating the linkages between service types and supplier relationships in servitized environments. Int J Prod Econ.

[CR86] Saghiri S, Wilding R (2021). On the effectiveness of supplier development programs: The role of supply-side moderators. Technovation.

[CR87] Sales-Vivó V, Gil-Saura I, Gallarza MG (2021). Comparing relationship of quality-satisfaction models: effects of B2B value co-creation. Int J Retail Distrib Manag.

[CR88] Sharma N (2021) How core, technical and social components of business relationship value drive customer satisfaction and loyalty in high tech B2B market. 10.1108/JBIM-12-2020-0554. J Bus Ind Mark ahead-of-p

[CR89] Shin N, Park S (2021). Supply chain leadership driven strategic resilience capabilities management: A leader-member exchange perspective. J Bus Res.

[CR90] Shnaiderman M, Ben-Baruch L (2016). Control and enforcement in order to increase supplier inventory in a JIT contract. Eur J Oper Res.

[CR91] Sila I, Ebrahimpour M, Birkholz C (2006). Quality in supply chains: an empirical analysis. Supply Chain Manag An Int J.

[CR92] Simon A, Kafel P (2018) Reasons for decertification of iso 9001. An empirical study. 10.15446/innovar.v28n70.74449. Innovar 28:

[CR93] Singh N (2014) Automotive Industry Response to Its Global QMS Standard ISO/TS-16949. pp 121–142

[CR94] Smith JR, Yost J, Lopez H (2020). Electronic data interchange and enterprise resource planning technology in supply chain contracts. Comput Ind Eng.

[CR95] Suh CJ, Kim J-H (2018). Buyers’ switching intentions in a manufacturing supply chain: a migration theory perspective. Int J Oper Prod Manag.

[CR96] Sun H, Cheng T-K (2002). Comparing Reasons, Practices and Effects of ISO 9000 Certification and TQM Implementation in Norwegian SMEs and Large Firms. Int Small Bus J Res Entrep.

[CR97] Sunil Kumar CV, Routroy S, Mishra RK (2018). Lean Supplier Management for Better Cost Structures. Mater Today Proc.

[CR98] Syreyshchikova NV, Pimenov DY, Mikolajczyk T, Moldovan L (2020). Automation of Production Activities of an Industrial Enterprise based on the ERP System. Procedia Manuf.

[CR99] Sytnik N, Kravchenko M (2021). Application of knowledge management tools: Comparative analysis of small, medium, and large enterprises. J Entrep Manag Innov.

[CR100] Taherdoost H, Brard A (2019). Analyzing the Process of Supplier Selection Criteria and Methods. Procedia Manuf.

[CR101] Tavakol M, Dennick R (2011). Making sense of Cronbach’s alpha. Int J Med Educ.

[CR102] Tavana M, Shaabani A, Di Caprio D, Amiri M (2021). An integrated and comprehensive fuzzy multicriteria model for supplier selection in digital supply chains. Sustain Oper Comput.

[CR103] Thabit TH (2021). The Extent of Applying ISO 14001 Requirements in the Environmental Auditing Practices of Iraq. J Tech.

[CR104] Tomic B, Spasojevic Brkic VK (2019). Customer satisfaction and ISO 9001 improvement requirements in the supply chain. TQM J.

[CR105] Tong X, Lai K, Zhu Q (2018). Multinational enterprise buyers’ choices for extending corporate social responsibility practices to suppliers in emerging countries: A multi-method study. J Oper Manag.

[CR106] Torabi SA, Baghersad M, Mansouri SA (2015). Resilient supplier selection and order allocation under operational and disruption risks. Transp Res Part E Logist Transp Rev.

[CR107] Tóth Z, Nieroda ME, Koles B (2020). Becoming a more attractive supplier by managing references – The case of small and medium-sized enterprises in a digitally enhanced business environment. Ind Mark Manag.

[CR108] Tseng M-L, Bui T-D, Lim MK (2022). Assessing data-driven sustainable supply chain management indicators for the textile industry under industrial disruption and ambidexterity. Int J Prod Econ.

[CR109] Upadhyay P, Basu R, Adhikary R, Dan PK (2010). A Comparative Study of Issues Affecting ERP Implementation in Large Scale and Small Medium Scale Enterprises in India: A Pareto Approach. Int J Comput Appl.

[CR110] Wang Y, Zhang L (2021). How customer entitlement influences supplier performance in B2B relationships in emerging economy? A moderated mediation model of institutional environments. J Bus Res.

[CR111] Wong CWY, Wong CY, Boon-itt S (2020). Environmental management systems, practices and outcomes: Differences in resource allocation between small and large firms. Int J Prod Econ.

[CR112] Yu Y, Zhang M, Huo B (2019). The impact of supply chain quality integration on green supply chain management and environmental performance. Total Qual Manag Bus Excell.

[CR113] Zakeri S, Chatterjee P, Cheikhrouhou N, Konstantas D (2022). Ranking based on optimal points and win-loss-draw multi-criteria decision-making with application to supplier evaluation problem. Expert Syst Appl.

[CR114] Zhan Y, Chung L, Lim MK (2021). The impact of sustainability on supplier selection: A behavioural study. Int J Prod Econ.

[CR115] Zhou J, Zhu J, Wang H (2021). Dual-sourcing and technology cooperation strategies for developing competitive supplier in complex product systems. Comput Ind Eng.

[CR116] Zimon D (2017). The influence of quality management systems for improvement of logistics supply in Poland. Oeconomia Copernicana.

